# Enriched Environment Prevents Surgery-Induced Persistent Neural Inhibition and Cognitive Dysfunction

**DOI:** 10.3389/fnagi.2021.744719

**Published:** 2021-10-01

**Authors:** Shana Yang, Song Zhang, Wenting Tang, Shunchang Fang, Hongyang Zhang, Jieyan Zheng, Xia Liu, Ying Zhang, Liang Zhao, Lianyan Huang, Boxing Li

**Affiliations:** ^1^Guangdong Provincial Key Laboratory of Brain Function and Disease, Neuroscience Program, Zhongshan School of Medicine and the Fifth Affiliated Hospital, Sun Yat-sen University, Guangzhou, China; ^2^Department of Spine Surgery, Changzheng Hospital, Naval Medical University, Shanghai, China; ^3^Department of Cardiology, Shanghai Chest Hospital, Shanghai Jiao Tong University, Shanghai, China

**Keywords:** neuroinflammation, neuronal activity, *in vivo* two-photon imaging, enriched environment, aging, perioperative neurocognitive disorders

## Abstract

Perioperative neurocognitive disorders (PND) encompass short-term delirium and long-term cognitive dysfunction. Aging increases the susceptibility to PND, yet the neural mechanism is not known. In this study, we monitored the dynamic changes of neuronal activity in the prelimbic cortex before and after surgery. We found that anesthesia combined with surgery, but not anesthesia alone, induced a prolonged decrease in neuronal activity during the post-operation period in the aged mice, but not in the adult mice. The prolonged decrease in neuronal activity was accompanied by surgery-induced microglial activation and proinflammatory cytokines expression. Importantly, we found that the enriched environment (EE) completely prevented both the prolonged neural inhibition and neuroinflammation, and improved cognitive function in the aged mice. These results indicate that the prolonged neural inhibition correlated to PND and that EE before the surgery could effectively alleviate the surgery- induced cognitive dysfunction.

## Introduction

Aging is one of the most severe challenges facing humankind. It causes the functional deterioration of many organs throughout the body, among which the brain is most severely affected (Yankner et al., 2008; Mattson and Arumugam, 2018). Aging induces significant changes in brain size and gross morphology and severely worsens brain function, especially cognitive and learning abilities (Burke and Barnes, 2006). This aging-related decline of cognitive function often results in a decrease in the quality of life. Although many hypotheses have been proposed regarding the mechanism of the aging-related cognitive impairment, the exact neural mechanism remains to be elucidated.

In addition to the decline in brain function caused by aging itself, aging will also reduce the brain’s adaptability, manifesting as a decrease in the ability to cope with external insults, which could, in turn, aggravate the impairments of cognitive function (Mattson and Arumugam, 2018). A typical example is the cognitive dysfunction that occurs after general anesthesia and surgery, the perioperative neurocognitive disorders (PND) (Bedford, 1955; Hanning, 2005; Eckenhoff et al., 2020). PND includes short-term delirium and long-lasting postoperative cognitive dysfunction. It is the most common complication experienced in the postoperative period by geriatric patients, with an incidence up to 50% within the first week after surgery and more than 10% after 3 months (Moller et al., 1998; Newman et al., 2007; Androsova et al., 2015; Inouye et al., 2016; Daiello et al., 2019). PND patients suffer from the decline of cognitive performance, including learning, and memory, attention and executive function, which leads to long-term disability, failure to return to work, a substantial increase in healthcare costs and higher mortality (Hanning, 2005; Steinmetz et al., 2009; Schulte et al., 2018; Eckenhoff et al., 2020). Because of the high incidence of PND and the severe consequence, it is urgent to understand the underlying neural mechanisms.

Many cellular and molecular events are thought to be closely related to PND. One of the most accepted mechanisms is neuroinflammation, including microglial activation and the release of cytokines and interleukins (Alam et al., 2018; Safavynia and Goldstein, 2018). Accordingly, the anti-inflammatory drugs and non-drug treatments were helpful for PND prevention by reducing neuroinflammation (Kawano et al., 2015; Safavynia and Goldstein, 2018). However, the direct and immediate effect of anesthetics on the brain is inhibiting neuronal activity. Would the immediate inhibition of the neuronal activity contribute to the susceptibility of the aged to PND? Could the intervention preventing this neuronal inhibition alleviate the cognitive dysfunction?

To answer these questions, we monitored the changes in the neuronal activity, presumed one of the earliest events during anesthesia and surgery, in the prelimbic cortex of the adult and aged mice using a transcranial two-photon Ca^2+^ imaging technique. We found that anesthesia combined with surgery, but not anesthesia alone, led to a significant and prolonged decrease in neuronal activity during the post-operation period in the aged mice but not in the adult mice. The prolonged decrease in neuronal activity was accompanied by surgery-induced microglial activation and proinflammatory cytokines expression. Importantly, we found that the enriched environment (EE), an convenient and widely used method for improving synaptic plasticity and neurological function (Sale et al., 2009; Sampedro-Piquero and Begega, 2017; Kempermann, 2019), could significantly ameliorate the abnormalities. It completely prevented the prolonged decrease in neuronal activity and surgery-related neuroinflammation and eventually improved cognitive function. Together, our results indicate that the prolonged decrease of neuronal activity is an early event correlated to PND and that physically and mentally enhancing neuronal activity before the surgery could effectively alleviate the surgery-induced cognitive dysfunction in PND.

## Materials and Methods

### Animals

Adult (3-month-old) and aged (14-month-old) C57BL/6 mice were provided by the Laboratory Animal Center of Sun Yat-sen University (Guangzhou, China). All the mice were group-housed 5 mice per cage under controlled conditions with the temperature at 25 ± 2°C and humidity at 50–60%. The lights were maintained in light-dark cycles of 12 h. The mice had free access to the SPF rodent diet and water. The study was approved by the Animal Care and Use Committee of Sun Yat-sen University.

### Laparotomy

The laparotomy was performed following previous studies (Rosczyk et al., 2008; Barrientos et al., 2012; Hovens et al., 2015). Briefly, the mice were anesthetized with propofol (160 mg/kg, lipid emulsion, intraperitoneal injection) to induce stable anesthesia without righting reflex or corneal reflex (Cattano et al., 2008). The mice were placed in warming pads and aseptic conditions and continuously observed for breathing and skin color. A midline laparotomy (∼4 cm in length) was performed. Sterile cotton dipped into sterile saline was used to explore in the following order: left kidney, right kidney, spleen, liver and bowel (about 10 min). Sterile saline was applied during the whole procedure to prevent drying. The muscle and the skin of the abdomen were closed with a 5.0 silk suture, respectively. To reduce the postoperative pain, we injected lidocaine (10 mg/kg) in the incision margins (Durst et al., 2021). During the procedure, the temperature of warming pads was maintained at 37°C. All surgical fields and instruments were maintained under aseptic conditions. The entire procedure usually took less than 30 min. After the surgery, the mice were placed in a warmed container and observed for several hours before returning to their home cage.

### Surgical Preparation for Imaging Awake, Head-Restrained Mice

The viral injection and surgical procedures were previously described (Huang et al., 2016, 2020). Briefly, AAV-CaMKIIα-GCaMP6s was diluted tenfold in ACSF for use. A total of 0.2 μl of AAV viruses were slowly injected over 15 min into Layer V pyramidal neurons of the prelimbic cortex using a glass microelectrode. Surgery preparation for awake animal imaging includes attaching the head to a specific holder and performing an open-skull cranial window 24 h before imaging. The head-specific holder was attached to the skull with cyanoacrylate-based glue to help restrain the animal’s head and reduce motion-related artifacts (animal’s respiratory rate) during imaging. A small skull region (∼0.2 mm in diameter) was located over the prelimbic cortex based on the stereotactic coordinate. A cranial window was created to image Layer V pyramidal neurons as previously described (Cichon and Gan, 2015; Bai et al., 2017). The skull surface was immersed in artificial cerebrospinal fluid, a high-speed drill and microsurgical forceps were used to carefully remove a small piece of skull overlying the prelimbic cortex of interest and replace it with a square glass coverslip (approximately the same size as the skull being removed). Glue to the skull to cover the exposed cortex and reduce brain motion. The entire surgical procedure usually took less than 15 min, and the two-photon imaging took place immediately after the surgical procedures.

### Two-Photon Calcium Imaging *in vivo*

The *in vivo* two-photon calcium imaging procedures were previously described (Huang et al., 2016, 2020). *In vivo* two-photon calcium imaging was performed with an Olympus FVMPE-RS system with the laser tuned to the optimal excitation wavelength for YFP (920 nm) equipped with a Deepsee Ti:Sapphire laser (MaiTai DeepSee, Spectra Physics). All experiments were performed using a 25 × water immersion objective (numerical aperture 1.1) immersed in an ACSF solution and with a 1.5 × digital zoom. The average laser power on the tissue sample was 10–40 mW for imaging to minimize phototoxicity. All the calcium signals were recorded at 2 Hz with a resolution of 512 × 512 pixels. The total imaging stack of each mouse consisted of 120 frames (about 1.6 min).

The neuronal activity was monitored at 5 time points: before anesthesia (baseline, –2 h), immediately after the anesthesia and surgery (0 h), 2 h after the anesthesia and surgery when the mice started to wake up and exhibited voluntary movements (2 h), 4 h (4 h), and 6 h (6 h) after the anesthesia and surgery.

### Imaging Data Analysis of Two-Photon Imaging

Image acquisition was analyzed using NIH ImageJ software as described previously (Bai et al., 2017; Lin et al., 2019). All imaging stacks were pre-processed using the ImageJ plugin stabilizer. The regions of interest (ROIs) were drawn manually around visually identifiable somas and then measured by averaging all pixels within each identifiable area above. The fluorescence change was calculated as ΔF/F0 = (F–F0)/F0, in which F0 was the average of 10% minimum *F*-values over a 1.6 min period, representing baseline fluorescence intensity after background subtraction. The threshold for determining calcium transients was more than three times the standard deviation (SD) plus the mean of fluorescence (ΔF/F0). The total calcium activity was the accumulated calcium activities during a 1.6 min period. The peak amplitude was the highest value of the calcium transient.

### Novel Object Recognition Test

The mice were habituated to the chamber (35 × 35 × 23 cm) and allowed to explore for 10 min without objects. Following habituation, mice were placed in the chamber with its head positioned opposite the two similar objects (towers of Lego bricks) for 10 min. After the familiar session, there was an intertrial interval (24 h), and one of the Lego bricks was replaced with a novel object (tissue culture flask). The mice were placed in the chamber freely for 5 min. Sniffing time that the mice spent with their nose oriented toward and within 2 cm of the object were measured by Topscan software (CleverSys Inc.). The preference was measured by the discrimination index, which was calculated as a percentage of time that mice spent with novel object compared with the total time exploring both objects.

### Fear Conditioning Test

Fear conditioning test was performed as previously described (Huang et al., 2017). The mice were habituated for 2 min in an enclosed chamber on an electrified wire floor or audible tones. The mice were then applied with three pairings of auditory cue (30 s, 400 Hz, 80 dB) co-terminating with a scrambled foot shock (2 s, 0.5 mA). The intertrial interval was 15 s. Then, the mice were returned to their home cage after 6 min fear conditioning training. The next day, fear contextual memory was measured by the percent of the time that the animals spent on freezing in response to the re-presentation of the context for 5 min without applying the shock. Fear auditory-cued memory was measured in a novel chamber different from the training day. The mice were habituated for 2 min and then the auditory cue (2 min, 400 Hz, 80 dB) was given without shock stimuli. The percentage of time that the animals spent on freezing during the tone presentation was recorded by the Freeze Frame fear conditioning system (Actimetrics Inc.).

### Enriched Environment

The EE cage consists of a polyethylene plate cage of 80 × 80 × 80 cm (10 animals per cage). The cage is equipped with two running wheels, plastic fitness balls, arch bridges and enriched with a complex of plastic toys, steel chains and tunnels in different sizes. Toys were replaced, and the tunnel shapes were changed every 3 days. The rest was rearranged inside the cage. The EE group mice were put in the EE cage for 4 weeks, and the sedentary group mice were in the same cage but in the absence of objects and running wheels. All cages were cleaned weekly.

### Quantitative Real-Time PCR Assay

The total RNA in the mPFC tissues of the C57BL/6 mice were extracted by SE Total RNA Kit I (Omega) according to the instructions. Reverse transcription-PCR was performed on a Prime-Script RT reagent Kit (Takara, Dalian, Liaoning, China) with gDNA eraser with 1 μg of total RNA according to the manufacturer’s instructions. The PCR primers were designed using Primer 5.0, and the sequences were listed below. The β-actin gene was served as an internal control. The reaction was carried out in 96-well plates with a total volume of 20 μl including 10 μl 2 × SYBR Premix Ex Taq (Takara, Dalian, Liao Ning, China), 6.2 μl ddH_2_O, 3 μl cDNA template and 0.4 μl forward and reverse primers (10 μM) respectively. The reactions were run on a Light Cycler 96 system (Roche) according to the manufacturer’s instructions. ΔCt and ΔΔCt values were calculated, and the gene expression values were calculated as 2-ΔΔCt.

TNF F:5′-CGTCAGCCGATTTGCTATCT-3′,
R:5′-CGGACTCCGCAAAGTCTAAG-3′;
IL-6 F:5′-ATGGATGCTACCAAACTGGAT-3′,
R:5′- TGAAGGACTCTGGCTTTGTCT-3′;
IL-1 F:5′-CCTTATTTCGGGAGTCTATTCA-3′,
R:5′- CTCCACTAGGGTTTGCTCTTCT -3′;
IL-1 F:5′- GCCCATCCTCTGTGACTCAT -3′,
R:5′- AGGCCACAGGTATTTTGTCG -3′.

### Immunofluorescence

Mice were sacrificed and perfused with 1 × PBS followed by 4% paraformaldehyde. The brain was removed and postfixed in 4% paraformaldehyde for 24 h. The temperature was maintained at 4°C. After immersed in 20% sucrose for 24 h, it was replaced with 30% sucrose for another 24 h. The brain was embedded in the optimal cutting temperature compound and stored at –80°C immediately. The brain was then sectioned coronally in 40 μm thick slices with a microtome (Leica, CM1950). For immunostaining, brain slices were washed 3 times with 1 × PBS and then permeabilized and blocked with blocking buffer (1 × PBS containing 0.25% Triton X-100 and 5% donkey serum) for 1 h and incubated with an anti-Iba1 antibody (rabbit, 1:500, WAKO, catalog #019-19741) for overnight (at 4°C). Immunostaining was performed with Alexa 555 fluorescent secondary antibody (donkey anti-rabbit, Invitrogen, A31572, 1:800) for 1 h at 37°C. Then, slices were counterstained with DAPI (Sigma) for 10 min and washed 3 times with 1 × PBS. A stack of images spanning brain slices from Iba1 stained mPFC slices was acquired using a confocal laser-scanning microscope (AxioScan.Z1) equipped with a 20 × (NA 1.25) objective. 3–5 slices from both sides of the mPFC were randomly selected from each animal. The number of Iba1^+^ cells (microglial cells) was counted manually due to the ramified morphology and small size of the soma. Briefly, a region of interest (504 × 510 pix) was selected, and the area was measured. The cells within the area were counted using NIH ImageJ software. Cell density was calculated as the number of Iba1^+^ cells divided by the total area of the chosen field (Favuzzi et al., 2021).

### Statistical Analysis

Statistical analyses were performed using GraphPad Prism (version 8.0). For two-group datasets, a two-tailed paired *t*-test or unpaired *t*-test was used when the dataset passed the normality test. Otherwise, a non-parametric test (Mann–Whitney test) was chosen. For more than two groups, one-way ANOVA or two-way ANOVA analyses were performed. Data are reported as mean ± SEM. A value of *P* ≤ 0.05 was considered statistically significant.

## Results

### Aged Mice Exhibited Increased Susceptibility to Perioperative Neurocognitive Disorders

**FIGURE 1:**
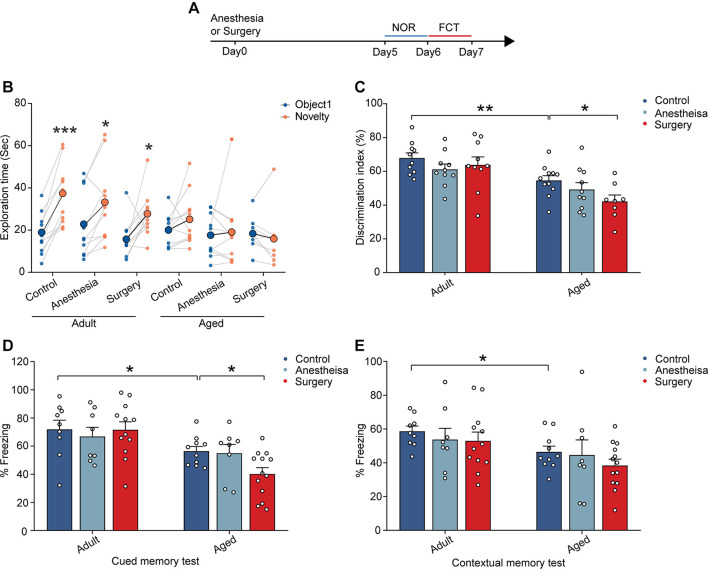
Aging-related cognitive impairments were aggravated by surgery. **(A)** Schematic outline of experimental approach. **(B)** The exploration time in NOR test. **(C)** The discrimination index in NOR test. **(D)** The percentage of freezing time in the auditory-cued memory test. **(E)** The percentage of freezing time in the contextual memory test. Data was presented as mean ± SEM. Data were analyzed by two-tailed Student’s *t*-test or One-way ANOVA followed by Tukey’s *post hoc* tests; *P < 0.05, **P < 0.01, ***P < 0.001.

In order to examine the effects of anesthesia and surgery on cognitive function, we assessed the learning and memory of the adult and aged mice by novel object recognition (NOR) test and fear conditioning test (FCT) after the administration of anesthesia (propofol) with or without surgery (laparotomy) (Figure 1A). In the control group (without anesthesia or surgery), the adult mice showed a significant preference for the novel object in the NOR test (*n* = 10, *P* = 0.0002; Figure 1B). In contrast, the aged mice did not show any apparent preference (Figure 1B). The discrimination index of the aged mice was significantly lower than that of the adult mice (aged vs. adult, 54.58 ± 2.80% vs. 67.84 ± 3.03%, *P* = 0.0045; Figure 1C). These results indicate that the aged mice exhibited cognitive dysfunction (Figures 1B,C). Strikingly, anesthesia combined with surgery (surgery thereafter), but not anesthesia alone (anesthesia thereafter), significantly worsened the performance of the aged mice. The discrimination index was even lower than that in the control group in the aged mice (aged surgery vs. aged control, 42.16 ± 3.78% vs. 54.58 ± 2.80%, *P* = 0.0431; Figure 1C). In contrast, anesthesia or surgery did not influence the performance of the adult mice (Figures 1B,C). These results indicate that surgery, but not anesthesia, could induce cognitive dysfunction in the aged mice and suggest that the aged mice were more vulnerable to cerebral insults to induce PND.

Similar results were also observed in the FCT. In contrast to the adult mice showing normal cued (71.92 ± 6.46%) and contextual (58.66 ± 3.10%) memory, the aged mice exhibited memory deficits with reduced freezing behaviors in auditory-cued test (56.44 ± 3.35%, *P* = 0.0424) and contextual memory test (46.48 ± 3.38%, *P* = 0.0173) (Figures 1D,E). Moreover, the administration of surgery, but not anesthesia, further reduced the freezing behaviors of the aged mice in the auditory-cued test (40.18 ± 4.52%, *P* = 0.0323) but not in the contextual test (Figures 1D,E). These results suggest that surgery aggravated aging-related cognitive impairments in the aged mice.

Together, these results indicate that, while aging led to cognitive dysfunction in the aged mice, the surgery, but not anesthesia, significantly aggravated the cognitive impairment in the aged mice.

### Aged Mice Exhibited a Prolonged Decrease in Neuronal Activity After Surgery

**FIGURE 2:**
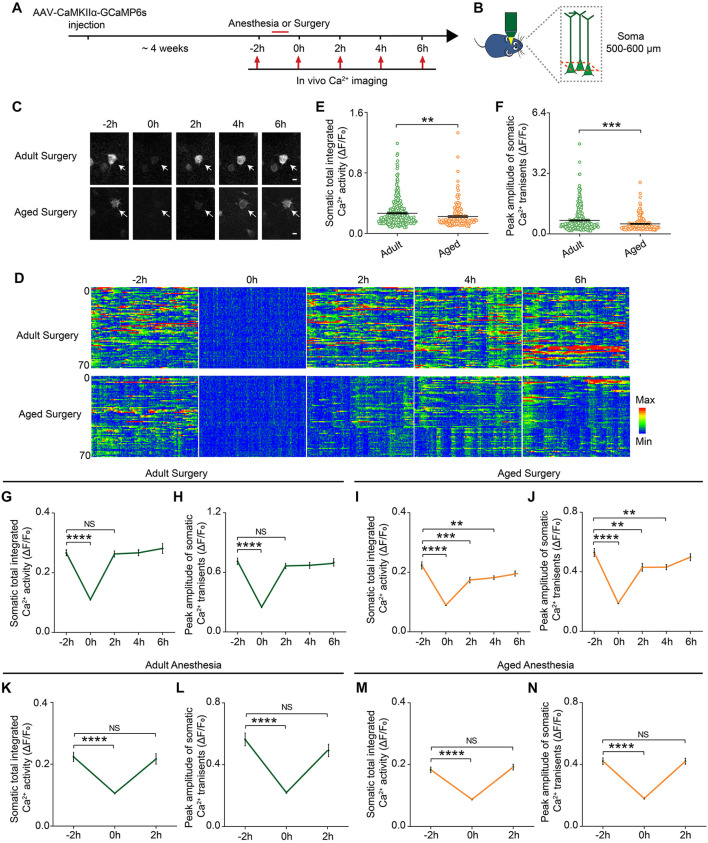
Prolonged decrease in neuronal activity in the aged mice after surgery. **(A)** Schematic timeline of two-photon calcium imaging in adult and aged mice. **(B)** Schematic illustrating the experimental approach for *in vivo* Ca^2+^ imaging in prelimbic cortex of mice. **(C)** Representative images of layer V pyramidal neurons expressing GCaMP6s. Scale bars: 10 μm. **(D)** Heatmaps of somatic Ca^2+^ activity of adult and aged mice at different time points (*n* = 70 cells in each group). **(E,F)** Quantification of somatic total integrated Ca^2+^ activity **(E)** and peak amplitude of somatic Ca^2+^ transients **(F)** before surgery (adult: *n* = 323 cells from 5 mice, aged mice: *n* = 169 cells from 5 mice). **(G,H)** Time course of changes in somatic total integrated Ca^2+^ activity **(G)** and peak amplitude of somatic Ca^2+^ transients **(H)** before and after surgery in adult mice (*n* = 323 cells from 5 mice). **(I,J)** Time course of changes in somatic total integrated Ca^2+^ activity **(I)** and peak amplitude of somatic Ca^2+^ transients **(J)** before and after surgery in aged mice (*n* = 169 cells from 5 mice). **(K,L)** Time course of changes in somatic total integrated Ca^2+^ activity **(K)** and peak amplitude of somatic Ca^2+^ transients **(L)** before and after anesthesia in adult mice (*n* = 79 cells from 3 mice). **(M,N)** Time course of changes in somatic total integrated Ca^2+^ activity **(M)** and peak amplitude of somatic Ca^2+^ transients **(N)** before and after anesthesia in aged mice (*n* = 126 cells from 4 mice). Data was presented as mean ± SEM. Data were analyzed by two-tailed Student’s *t*-test or one-way ANOVA followed by Tukey’s *post hoc* tests; **P < 0.01, ***P < 0.001, ****P < 0.0001, NS stands for No Significant.

Neuronal activity is critical for brain functions. To investigate the early impacts of anesthesia and surgery on the brain, we monitored the neuronal activity, presumed one of the earliest changes induced by the anesthesia and surgery, in the prelimbic cortex in the adult and aged mice (Figure 2A). AAV expressing genetically encoded Ca^2+^ indicator GCaMP6s under CaMKIIα promoter was injected into the layer V pyramidal neurons in the prelimbic cortex. Four weeks after the injection, the neuronal activity before and after the administration of anesthesia or surgery was monitored by transcranial two-photon Ca^2+^ imaging (see section “Materials and Methods”) (Figures 2A,B). The results showed that, before the administration of anesthesia or surgery (–2 h), the aged mice showed much less spontaneous neuronal activity than the adult mice (Figures 2C,D). The total integrated Ca^2+^ activity and peak amplitude of Ca^2+^ transients in the aged mice were significantly lower than those in the adult mice (*P* < 0.0001; Figures 2E,F).

During the administration of surgery (0 h), the neuronal activity in both adult and aged mice was significantly suppressed by the anesthetics (propofol) (*P* < 0.0001; Figures 2C–J). After surgery, the neuronal activity of the adult mice fully recovered to the pre-operation level once the mice started to wake up and exhibited voluntary movements (2 h) (Figures 2G,H). However, in the aged mice, the neuronal activity remained significantly lower than the pre-operation level when the mice woke up from the anesthesia and exhibited voluntary movements. Both total integrated Ca^2+^ activity and peak amplitude of Ca^2+^ transients were not fully restored until 6 h after surgery (Figures 2I,J). These results indicate that surgery led to a prolonged decrease in neuronal activity in the aged mice.

We also examined the dynamic changes of the neuronal activity before and after the administration of anesthesia alone. Although propofol injection also instantly suppressed the total integrated Ca^2+^ activity and peak amplitude of Ca^2+^ transients (*P* < 0.0001), the neuronal activity of both adult and aged mice recovered rapidly after the animals woke up and exhibited voluntary movements (Figures 2K–N).

Together, these results indicate that surgery, but not anesthesia alone, could induce a prolonged decrease in neuronal activity in the aged mice.

### Surgery Exacerbated Delayed Aging-Related Neuroinflammation

**FIGURE 3:**
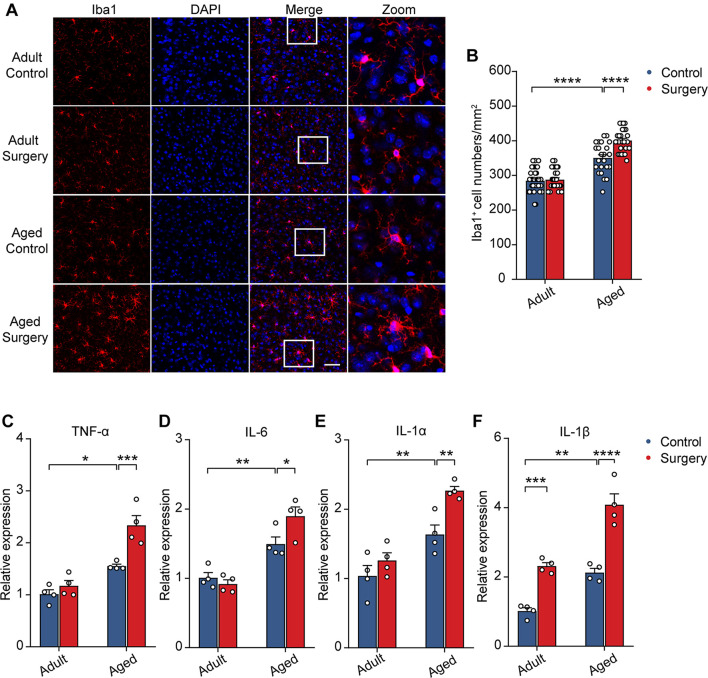
Surgery exacerbated aging-related neuroinflammation. **(A)** Representative images of Iba1^+^ microglia in mPFC of the adult and aged mice. Scale bars: 50 μm. **(B)** Quantification of Iba1^+^ cell numbers in each group. **(C–F)** Expression level of TNF-α **(C)**, IL-6 **(D)**, IL-1α **(E)** and IL-1β **(F)** in each group (*n* = 4 mice). Data was presented as mean ± SEM. Data was analyzed by two-way ANOVA and Bonferroni’s correction. *P < 0.05, **P < 0.01, ***P < 0.001, ****P < 0.0001.

Previous studies showed that neuronal activity profoundly influenced microglia function (Nimmerjahn et al., 2005; Wake et al., 2009; Eyo et al., 2014, 2016). The reduction of neuronal activity also led to enhanced microglia process surveillance *in vivo* (Liu et al., 2019). Given the findings that microglia would develop a more inflammatory phenotype in the aging brain (Kyrkanides et al., 2001; Sparkman and Johnson, 2008; Damani et al., 2011; Harry, 2013; Norden et al., 2015) and that the administration of surgery procedure induced a prolonged decrease in neuronal activity (Figure 2), we hypothesized that surgery would exaggerate the activation of microglia in the aged brain. To test this, we first compared the microglial activation in the adult and aged mice with or without surgery. The number of Iba1 (the marker for activated microglia) positive cells in the adult mice was comparable between the control and surgical groups (Figures 3A,B). Our results showed that aging significantly increased the density of Iba1 positive microglia (aged vs. adult, 350.30 ± 9.26 vs. 284.57 ± 5.75, *P* < 0.0001; Figures 3A,B). Strikingly, surgery significantly elevated the number of Iba1 positive microglia in the aged mice (400.69 ± 6.43, *P* < 0.0001; Figures 3A,B), suggesting that microglial activation was exaggerated after surgery in the aged mice.

Further, we examined the mRNA expression of the proinflammatory cytokines in the adult or aged brains with or without surgery. Our results showed that, without surgery, the expression of TNF-α, IL-6, IL-1α, and IL-1β was higher in the aged mice than in adult mice, implying the neuroinflammation in the aged brain (aged vs. adult, TNF-α: 1.55 ± 0.04 vs. 1.01 ± 0.08, *P* = 0.0134; IL-6: 1.49 ± 0.10 vs. 1.01 ± 0.07, *P* = 0.0079; IL-1α: 1.64 ± 0.14 vs. 1.04 ± 0.15, *P* = 0.0085; IL-1β: 2.12 ± 0.12 vs. 1.01 ± 0.10, *P* = 0.0020). After surgery, in the adult mice, the mRNA expression of TNF-α, IL-6, and IL-1α was not changed, but the mRNA expression of IL-1β was increased (Figures 3C–F). In contrast, in the aged mice, the mRNA expression of TNF-α, IL-6, IL-1α, and IL-1β was all significantly increased after surgery (Figures 3C–F) (aged surgery vs. aged control, TNF-α: 2.34 ± 0.19 vs. 1.55 ± 0.04, *P* = 0.0006; IL-6: 1.90 ± 0.13 vs. 1.49 ± 0.10, *P* = 0.0244; IL-1α: 2.27 ± 0.06 vs. 1.64 ± 0.14, *P* = 0.0059; IL-1β: 4.08 ± 0.31 vs. 2.12 ± 0.12, *P* < 0.0001).

Together, these results suggest that surgery exacerbated aging-related neuroinflammation in aged mice.

### Enriched Environment Prevented the Surgery-Induced Prolonged Decrease in the Neuronal Activity in Aged Mice

**FIGURE 4:**
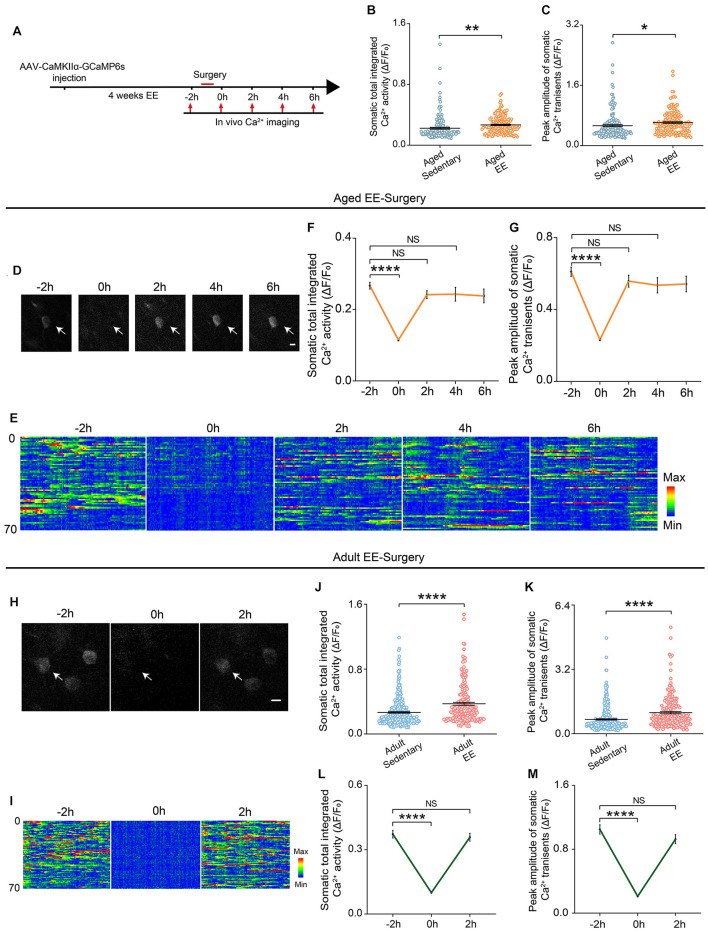
EE prevented the prolonged reduction of neuronal Ca^2+^ activity induced by surgery in the aged mice. **(A)** Schematic of experimental design. **(B,C)** Quantification of somatic total integrated Ca^2+^ activity **(B)** and peak amplitude of somatic Ca^2+^ transients **(C)** (*n* = 169 cells from 5 mice for aged sedentary group from Figure 2, *n* = 149 cells from 4 mice for aged EE group). **(D)** Representative images of layer V pyramidal neurons expressing GCaMP6s in aged EE-Surgery mice. Scale bars: 10 μm. **(E)** Heatmaps of somatic Ca^2+^ activity of the aged EE-Surgery mice at different time points (*n* = 70 cells in each group). **(F,G)** Time course of changes in somatic total integrated Ca^2+^ activity **(F)** and peak amplitude of somatic Ca^2+^ transients **(G)** before and after surgery in aged EE-Surgery mice (*n* = 149 cells from 4 mice). **(H)** Representative images of layer V pyramidal neurons expressing GCaMP6s in adult EE-Surgery mice. Scale bars: 10 μm. **(I)** Heatmaps of somatic Ca^2+^ activity of the adult EE-Surgery mice at different time points (*n* = 70 cells). **(J,K)** Quantification of somatic total integrated Ca^2+^ activity **(J)** and peak amplitude of somatic Ca^2+^ transients **(K)** before surgery (*n* = 323 cells from 5 mice for adult sedentary group from Figure 2, *n* = 193 cells from 5 mice for adult EE group). **(L,M)** Time course of changes in somatic total integrated Ca^2+^ activity **(L)** and peak amplitude of somatic Ca^2+^ transients **(M)** before and after surgery in adult EE-Surgery mice (*n* = 193 cells from 5 mice). Data was presented as mean ± SEM. NS stands for No Significant. Data was analyzed by One-way ANOVA followed by Tukey’s *post hoc* tests. *P < 0.05, **P < 0.01, ****P < 0.0001.

Previous studies have shown that the EE was an effective way to enhance animal’s space exploration, cognitive activities and physical exercise. We reasoned that these enhancements might prevent the prolonged decrease in neuronal activity induced by surgery in the aged mice. To test this, we placed the aged mice in a cage with EE for 4 weeks before surgery and examined the neuronal activity before and after surgery (Figure 4A). Our results showed that both total integrated Ca^2+^ activity and peak amplitudes of Ca^2+^ transients were increased after EE treatment (Figures 4B,C). Strikingly, although EE did not change the time to wake up and exhibit voluntary movements in the aged mice, EE significantly shortened the recovery time of the neuronal activity (Figures 4D,E). Both total integrated Ca^2+^ activity and peak amplitudes of Ca^2+^ transients were restored to pre-operation level when the mice woke up (Figures 4F,G) (Total integrated Ca^2+^ activity: –2 h, 0.27 ± 0.01; 2 h, 0.24 ± 0.01; 4 h, 0.24 ± 0.02. Peak amplitudes: –2 h, 0.61 ± 0.03; 2 h, 0.56 ± 0.03; 4 h, 0.53 ± 0.04).

We also examined the effects of EE on neuronal activity in adult mice (Figures 4H,I). Our results showed that 4-weeks treatment of EE before surgery also elevated the total integrated Ca^2+^ activity and peak amplitudes of Ca^2+^ transients (Figures 4J,K). However, EE did not change the recovery time of the neuronal activity after surgery in the adult mice (Figures 4H,I). Both total integrated Ca^2+^ activity (2 h vs. –2 h, 0.36 ± 0.02 vs. 0.37 ± 0.02, *P* = 0.7417) and peak amplitudes of Ca^2+^ transients (2 h vs. –2 h, 0.93 ± 0.06 vs. 1.05 ± 0.06, *P* = 0.1248) were restored to pre-operation level within 2 h when the mice woke up (Figures 4L,M).

These results suggest that EE could elevate neuronal activity in both adult and aged mice and effectively prevent the surgery-induced prolonged decrease in neuronal activity in the aged mice.

### Enriched Environment Prevented the Exaggerated Neuroinflammation Induced by Surgery in the Aged Mice

**FIGURE 5:**
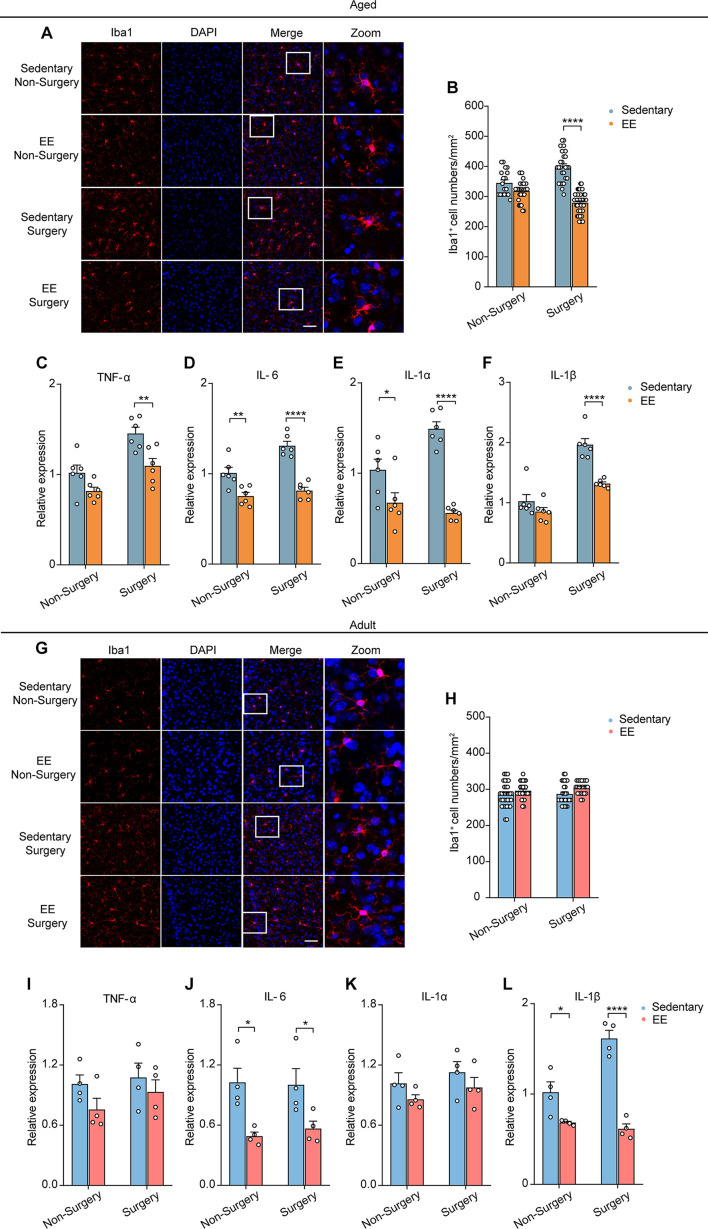
EE prevented the exaggerated neuroinflammation induced by surgery in the aged mice. **(A)** Representative images of Iba1^+^ microglia in mPFC of the aged mice in each group. Scale bars: 50 μm. **(B)** Quantification of Iba1^+^ cell numbers in each group of the aged mice. **(C–F)** Expression level of TNF-α **(C)**, IL-6 **(D)**, IL-1α **(E)** and IL-1β **(F)** in each group (*n* = 6 mice). **(G)** Representative images of Iba1^+^ microglia in mPFC of the adult mice in each group. Scale bars: 50 μm. **(H)** Quantification of Iba1^+^ cell numbers in each group for the adult mice. **(I–L)** Expression level of TNF-α **(I)**, IL-6 **(J)**, IL-1α **(K)** and IL-1β **(L)** in each group (*n* = 4 mice). Data was presented as mean ± SEM. Data was analyzed by two-way ANOVA and Bonferroni’s correction; *P < 0.05, **P < 0.01, ****P < 0.0001.

Next, we investigated EE’s effects on the activation of microglia and the expression of proinflammatory cytokines. We first examined the Iba1 positive microglia in mPFC of the aged mice in the non-surgery and surgery groups, with or without 4 weeks EE treatment. Notably, while EE did not change the microglial activation in the non-surgery group, EE completely prevented the elevation of microglial activation in the surgery group (EE vs. sedentary, 279.38 ± 5.99 vs. 398.61 ± 9.91, *P* < 0.0001; Figures 5A,B). Further, we examined the effects of EE on the mRNA expression of the proinflammatory cytokines after surgery. Our results showed that 4 weeks EE treatment significantly reduced the mRNA expression level of TNF-α, IL-6, IL-1α, and IL-1β in the surgery group of the aged mice (Figures 5C–F) (EE vs. sedentary, TNF-α, 1.10 ± 0.08 vs. 1.45 ± 0.07, *P* = 0.0041; IL-6, 0.81 ± 0.04 vs. 1.31 ± 0.05, *P* < 0.0001; IL-1α, 0.56 ± 0.03 vs. 1.49 ± 0.08, *P* < 0.0001; IL-1β, 1.32 ± 0.03 vs. 1.96 ± 0.10, *P* < 0.0001). Notably, EE could even reduce the expression level of IL-6 (*P* = 0.0024) and IL-1α (*P* = 0.021), but not that of TNF-α and IL-1β, in the non-surgery group of the aged mice (Figures 5D,E).

In contrast, in the adult mice, EE did not change the number of Iba1 positive microglia in either the non-surgery or surgery group (Figures 5G,H). However, for the expression of proinflammatory cytokines, EE had no effects on the mRNA expression of TNF-α and IL-1α but significantly reduced the mRNA expression of IL-6 and IL-1β in both non-surgery and surgery groups of the adult mice (Figures 5I–L) (EE vs. sedentary: IL-6, 0.49 ± 0.04 vs. 1.03 ± 0.14, *P* = 0.0130; IL-1β, 0.68 ± 0.01 vs. 1.02 ± 0.12, *P* = 0.0210. EE surgery vs. sedentary surgery: IL-6, 0.57 ± 0.07 vs. 1.00 ± 0.16, *P* = 0.040; IL-1β, 0.61 ± 0.05 vs. 1.62 ± 0.09, *P* < 0.0001). These results indicate that, besides preventing the early prolonged decrease in neuronal Ca^2+^ activity, EE could also ameliorate the delayed surgery-induced exaggeration in neuroinflammation in the aged mice.

### Enriched Environment Alleviated the Surgery-Induced Cognitive Dysfunction in the Aged Mice

**FIGURE 6:**
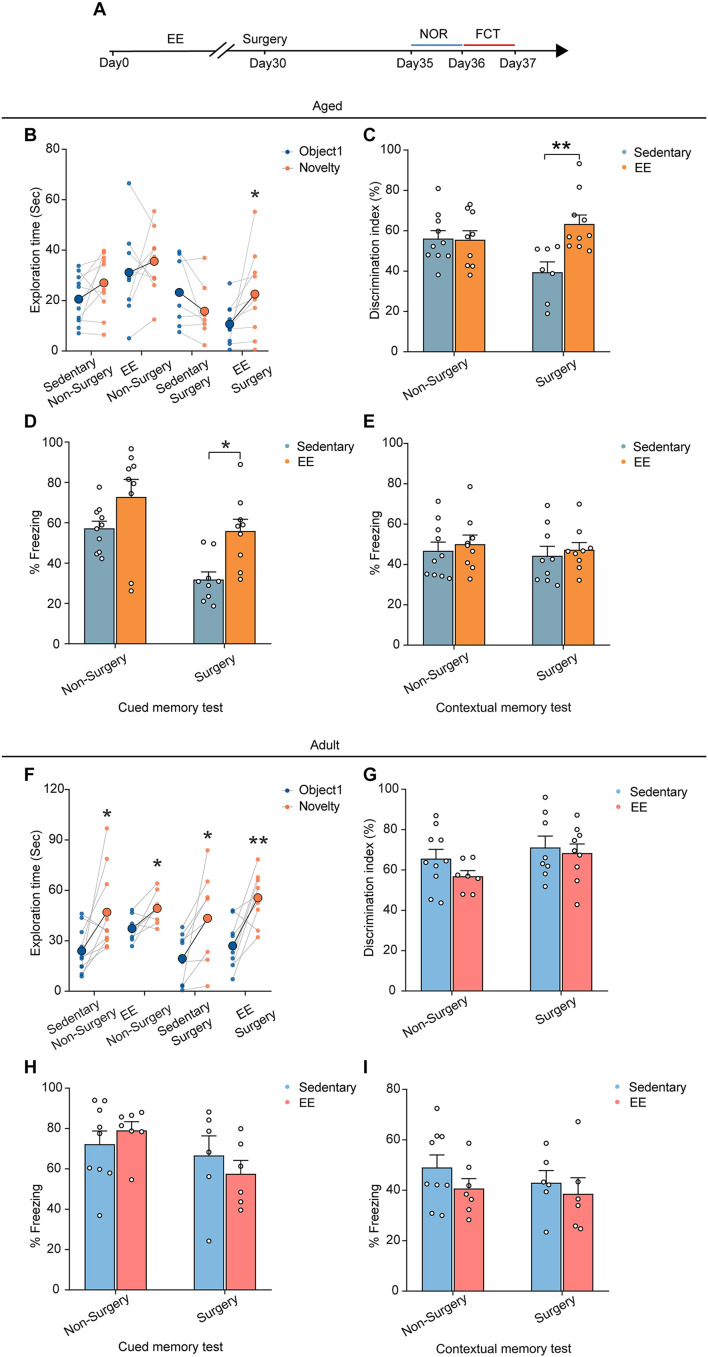
EE alleviated the cognitive dysfunction induced by surgery in the aged mice. **(A)** Schematic outline of experimental approach. **(B)** The exploration time in NOR test of the aged mice. **(C)** The discrimination index in NOR test of the aged mice. **(D)** The percentage of freezing time in the auditory-cued memory test of the aged mice. **(E)** The percentage of freezing time in the contextual memory test of the aged mice **(F)** The exploration time in NOR test of the adult mice. **(G)** The discrimination index in NOR test of the adult mice. **(H)** The percentage of freezing time in the auditory-cued memory test of the adult mice. **(I)** The percentage of freezing time in the contextual memory test of the adult mice. Data was presented as mean ± SEM. Data were analyzed by two-tailed Student’s paired *t*-test or two-way ANOVA and Bonferroni’s correction; *P < 0.05, **P < 0.01.

Finally, we asked whether EE could prevent the cognitive deficits induced by the surgery in the aged mice. We placed the aged mice in a cage with EE for 4 weeks before surgery and examined the cognitive function with NOR and FCT after the surgery (Figure 6A). Our results showed that, while EE did not improve the preference for the novel object in the NOR test in the non-surgery group of the aged mice, EE significantly improved the performance in the surgery group, increasing the discrimination index comparable to that of the non-surgery group (∼60%) (EE vs. sedentary, 63.34 ± 4.47% vs. 39.44 ± 5.12%, *P* = 0.0020; Figures 6B,C). These results suggest that while EE could not prevent aging-induced cognitive dysfunction in the aged mice, EE completely prevented the aggravation of cognitive impairment induced by the surgery.

Similarly, in the FCT, EE did not alter the freezing behaviors in both the contextual memory test and auditory-cued test in the non-surgery group (Figures 6D,E). However, EE significantly improved the performance in the auditory-cued test in the surgery group (EE vs. sedentary, 55.91 ± 5.86% vs. 31.84 ± 3.78%, *P* = 0.0125), leaving the performance in the contextual memory unchanged (Figures 6D,E).

To test whether EE has similar effects on the performance of the adult mice, we treated the adult mice with 4 weeks EE before surgery and examined the cognitive function. Our results showed that EE did not alter the performance in either test compared to the sedentary group. The discrimination index in the NOR test and the freezing behaviors in the FCT were comparable in both non-surgery and surgery groups (Figures 6F–I).

Together, these results suggest that although EE was not an effective way to improve the cognitive function in adult and aged mice, EE completely prevented the aggravation of cognitive impairment induced by the surgery in the aged mice.

## Discussion

Identifying the effects of aging on cognitive function and increased susceptibility to diseases is the primary focus of neuroscience and aging studies. In this study, we found that aging led to cognitive dysfunction and that surgery severely aggravated the cognitive impairment in the aged mice. Mechanistically, surgery induced a prolonged decrease in neuronal activity in the aged mice. Furthermore, the prolonged decrease of neuronal activity was accompanied by delayed neuroinflammation, including microglial activation and proinflammatory cytokine expression. Importantly, we found that EE before the surgery completely prevented the early prolonged decrease in the neuronal activity and the delayed activation of neuroinflammation and eventually alleviated the surgery-induced cognitive dysfunction in the aged mice.

The aging brain is more susceptible to neural diseases, yet the underlying mechanism is unknown. Consistent with our previous study on motor cortex, our results showed that spontaneous neuronal activity in the prefrontal cortex was significantly lower in the aged mice than in the adult mice (Huang et al., 2020). The reduction of neuronal activity would lead to the abnormality in the intracellular Ca^2+^ signaling that is important for properly adapting the external environmental changes and insults in the brain. Indeed, our results showed that, after surgery, the aging brain displayed a much worse response with a prolonged decrease in neuronal activity, whereas the adult brain recovered much sooner. Thus, the aging in the brain is essentially a decline in the brain’s ability to cope with external changes. This decline in adaptation ability may be attributed to the aging- related molecular changes in the brain because many neuronal activity- and synaptic activity-related proteins, such as calmodulin, glutamate receptors and synaptophysin, were decreased during aging (Smith et al., 2000; Lu et al., 2004; Magnusson et al., 2007; Morrison and Baxter, 2012).

Whether anesthetics alone could induce cognitive dysfunction or not is still controversial. It depends on the anesthesia type, the concentration and duration of anesthesia exposure and the animal’s pre-existing conditions (Belrose and Noppens, 2019). Propofol is an intravenous general anesthetic, potentiating gamma-aminobutyric acid receptors (Trapani et al., 2000). In our study, propofol administration alone did not cause the behavioral deficits and the prolonged decrease in the neuronal activity in the aged mice, suggesting that shorter exposures and lower doses of propofol may not cause cognitive dysfunction in aged mice. Our results suggested that the prolonged decrease in neuronal activity might be a primary contributor to the aging-related vulnerability to PND. Several factors could contribute to this prolonged decrease in the neuronal activity in the aged brain, including the insufficient clearance of the anesthetic from the brain, higher affinity of the anesthetic to the receptors and slower recovery of the intrinsic excitability. However, the exact mechanism needs further investigation.

The prolonged decrease in neuronal activity could trigger many downstream events in the brain. On the one hand, this prolonged decrease in neuronal activity would induce the inhibition of Ca^2+^ signaling pathways in the neurons. This would result in the suppression of Ca^2+^-dependent gene expression involved in synaptic plasticity and neuronal functions and eventually lead to cognitive dysfunction (Deisseroth et al., 2003; Li et al., 2016). On the other hand, the prolonged decrease in neuronal activity would influence microglial functions. Recent studies showed that neuronal activity profoundly impacts microglia process motility (Nimmerjahn et al., 2005; Wake et al., 2009; Eyo et al., 2014, 2016; Liu et al., 2019). The increase in the neuronal activity could inhibit the motility of the microglia process, whereas the decrease in the neuronal activity could lead to the higher motility of the microglia process (Liu et al., 2019; Merlini et al., 2021). Consistent with these findings, our results showed that the prolonged decrease in neuronal activity was accompanied by a delayed microglial activation and excessive release of the proinflammatory cytokines in the aged brain. The microglial activation would, in turn, aggravate the defeated neuronal function in the aged brain. This detrimental feedback loop then eventually led to PND in the aged mice. Furthermore, we noticed that EE had no effect on Iba1^+^ cell number but reduced IL-6 and IL-1β in adult mice. This implies that the expression of Iba1 and proinflammatory cytokines had distinct regulatory mechanisms (Xiao et al., 2004; Wongchana and Palaga, 2012; Maurya and Mishra, 2017, 2018; Zhang et al., 2018).

Our study suggests that EE could be an effective way to prevent the aggravation of cognitive impairment induced by the surgery in the aged mice. EE enhances exploration, social interaction and physical exercise, which improve physical, psychological, or emotional conditions (Sale et al., 2009; Sampedro-Piquero and Begega, 2017; Kempermann, 2019). In addition, much evidence shows that EE effectively enhances learning and memory in various tasks (Kobayashi et al., 2002; Sale et al., 2009; Sampedro-Piquero and Begega, 2017). We have previously shown that enriched motor experience effectively improved both learning-induced dendritic spine plasticity and motor performance in adult mice with early exposure to general anesthesia (Huang and Yang, 2015). EE was also shown to have beneficial effects on age- and operation-related changes (Rampon et al., 2000; Briones et al., 2013; Kawano et al., 2015; Ali et al., 2019; Kempermann, 2019). Consistent with previous findings (Kawano et al., 2015), our results showed that EE completely prevented the aggravation of cognitive impairment induced by the surgery in the aged mice, but did not improve the cognitive function of adult mice and aged mice without surgery. These results give us two clues in understanding the underlying mechanisms: (1) The beneficial effects of EE were unlikely due to the mere increase in neuronal activity before surgery, because EE also increased the neuronal activity in the adult mice, yet did not lead to the cognitive improvement (Figures 4, 6); (2) Preventing cognitive dysfunction and improving normal cognitive function might rely on distinct mechanisms. Importantly, we found that EE effectively prevented the early prolonged neuronal inhibition and broke the detrimental feedback loop in the neuron-microglia interaction, providing a new perspective to understand the role of EE in PND.

There are several limitations in this study. First, the molecular mechanisms of how EE affected neuronal activity and regulated microglial activation were still unknown. The EE protocol in our study consisted of both environment (toys, balls, and steel chains) and voluntary exercise (running wheels, bridges, and tunnels) components. Both components might contribute to the beneficial effects of EE on cognitive function, yet their distinct effects need to be elucidated. Second, we only examined the number of Iba1^+^ cells and the expression of proinflammatory cytokines. Whether other key features of microglial activation, such as the motility and morphology, were altered, and whether the level of proinflammatory cytokines in the serum contributes to the neuroinflammation will be investigated in future studies to understand the effects of EE on neuroinflammation comprehensively. Finally, similar to most animal studies, the clinical translation of our findings should be cautious as many neuronal functions are substantially different between humans and mice.

## Data Availability Statement

The raw data supporting the conclusions of this article will be made available by the authors, without undue reservation.

## Ethics Statement

The animal study was reviewed and approved by the Institutional Animal Care and Use Committee of Sun Yat-sen University. Written informed consent was obtained from the owners for the participation of their animals in this study.

## Author Contributions

BL and LH conceived the project. SY performed two-photon imaging. SZ and HZ performed immunostaining. SZ, WT, and XL performed qRT-PCR. SY and SZ performed behavioral tests. SY, SZ, JZ, YZ, LZ, and SF analyzed data. BL, LH, and SY wrote the manuscript. All authors contributed to the article and approved the submitted version.

## Conflict of Interest

The authors declare that the research was conducted in the absence of any commercial or financial relationships that could be construed as a potential conflict of interest.

## Publisher’s Note

All claims expressed in this article are solely those of the authors and do not necessarily represent those of their affiliated organizations, or those of the publisher, the editors and the reviewers. Any product that may be evaluated in this article, or claim that may be made by its manufacturer, is not guaranteed or endorsed by the publisher.
